# Phylodynamic and phylogeographic reconstruction of IBV lineages: diverse paths and determinants, one goal for control

**DOI:** 10.1038/s41598-025-21138-8

**Published:** 2025-10-23

**Authors:** Giovanni Franzo, Matteo Legnardi, Riccardo Baston, Francesca Poletto, Cristina Andolfatto, Claudia Maria Tucciarone, Mattia Cecchinato

**Affiliations:** Department of Animal Medicine, Production and Health (MAPS), Padua University, Legnaro, 35020 Italy

**Keywords:** IBV, Phylodynamic, Phylogeography, Epidemiology, Spreading factors, GLM, Diseases, Ecology, Ecology

## Abstract

Infectious bronchitis virus (IBV) is a genetically diverse avian coronavirus that significantly impacts global poultry production. While IBV evolution and spread have been widely studied, most analyses lack global scope and statistical evaluation of dispersal drivers. This study investigates the population dynamics, spatiotemporal diffusion, and determinants of spread for three major IBV lineages—GI-16, GI-19, and GI-23—using a phylodynamic framework integrated with a generalized linear model (GLM). All lineages likely originated between the 1960s and 1980s, circulating at low levels before expanding under favorable conditions, often linked to introduction into naïve or poorly managed poultry systems. Temporal trends suggested lineage competition and modulation by vaccination strategies. Phylogeographic analyses showed that local and regional circulation prevailed over long-distance migration. No statistical support was found for a role of migratory birds. Instead, chicken population size, agricultural investment and trade, were significant drivers. Interestingly, agricultural investment was negatively associated with viral migration in Europe—likely due to the protective effect of improved biosecurity and vaccination—yet positively associated with GI-23, reflecting rapid poultry sector expansion in some emerging economies, which was not matched by adequate animal health management. These findings highlight the complex and lineage-specific nature of IBV spread and the importance of science-based, coordinated control strategies, backed by international surveillance.

**Supplementary Information:**

The online version contains supplementary material available at 10.1038/s41598-025-21138-8.

## Introduction

Infectious bronchitis (IB) represents a major cause of economic losses in the global poultry industry. It is caused by infectious bronchitis virus (IBV), a coronavirus belonging to the species *Gammacoronavirus galli*, genus *Gammacoronavirus* (https://ictv.global/taxonomy). The infection is typically associated with mild to moderate respiratory signs, reproductive disorders, and decreased egg production. However, more severe clinical outcomes and increased mortality may occur with nephropathogenic strains or in the presence of secondary infections^1,2^. Additional costs are related to its control, which relies primarily on biosecurity measures, appropriate farm management, and vaccination strategies. The viral genome, approximately 27 kb in length, encodes both non-structural (such as the RNA-dependent RNA polymerase (RdRp) and other accessory and regulatory proteins) and structural proteins (i.e., the spike, envelope, membrane, and nucleocapsid proteins)^3^. Similarly to other coronaviruses, IBV is characterized by a high substitution rate (~ 10⁻³–10⁻⁴ substitutions/site/year)^4^ and a remarkable propensity for recombination^5,6^, both of which have contributed to its considerable genetic and phenotypic diversity over time. Although the entire genome is subject to variation, the spike (S) gene is by far the most extensively studied due to its critical biological functions. It mediates viral attachment to host cells, thereby determining host and tissue tropism. Moreover, it represents the main target of the host immune response, which further drives its diversification^7^. Consequently, the spike gene, or specific subregions, is most frequently sequenced for molecular epidemiological analyses and classification purposes. IBV, is currently classified in genotypes and lineages based on the phylogenetic analysis of the S1 gene and comparison with reference strains^8^. Some of those lineages showed a limited geographical distribution and/or temporal persistence, while others reached a worldwide distribution^9–11^.

One of the most relevant practical implications of IBV genetic variability is the limited cross-protection among distantly related strains, which undermines the effectiveness of pre-existing immunity, including vaccine-induced protection^12,13^. Although vaccines derived from different lineages have been developed —and the combined use of vaccines based on distinct variants has proven effective in broadening the spectrum of cross-protection—the introduction of novel variants evading population immunity remains a major concern^14,15^. Several studies have demonstrated IBV ability to spread both locally and internationally^4,16–18^, further complicating control efforts. Animal movements, socio-economic and cultural exchanges among countries, and, more recently, the role of migratory birds have been suggested as potential drivers or enhancers of viral dissemination^4,19–22^.

However, in all such instances, conclusions have been primarily based on subjective interpretations of genetic similarity among strains sampled in different countries or, at best, on the evaluation of migration events inferred through phylogeographic reconstructions. To date, no formal statistical analysis has been conducted to assess the relative contribution of different potential determinants to viral spread. The present study aims to address this gap by modelling the inferred migration rates between countries as a function of several potential predictors. These are formally tested using a generalized linear model (GLM) within the framework of a Bayesian discrete-state phylogeographic approach, thus allowing for robust statistical evaluation.

## Results

### Sequence dataset

According to the established inclusion criteria, lineages GI-16, GI-19, and GI-23 were selected for analysis. Initially, 132 (spike region 893–1240), 3386 (spike region 846–1249), and 795 (spike region 813–1241) sequences covering the hypervariable region 3 (HVR3), as defined according to previously published consensus criteria and further confirmed through comparison with sequences obtained from the same genomic region, were classified within these groups. However, after applying quality filters, genetic region selection, removing recombinant and vaccine-like strains, 112, 2739, and 271 sequences were retained, originating from 13, 23, and 16 countries and collected since 1983, 1993, and 1998, respectively.

Finally, to ensure computational feasibility given the large number of GI-19 sequences, this dataset was randomly down-sampled by selecting up to 10 sequences per country-year pair, obtaining a final dataset of 626 sequences.

Additionally, due to the recent emergence of GI-23 in the Americas^23^ and ongoing debate regarding the origin of this introduction—a separate analysis was performed for GI-23 selecting the genetic region corresponding to the other hypervariable regions 1 and 2 (HVR12) (from 14 countries, starting from 1998), that was the most used in South American countries^23,24^, although allowing for a lower resolution in the Middle East (Table 1).

Finally, a dataset including only European GI-19 sequences was generated since the absence of trade restrictions should theoretically facilitate inter-country exchanges. Additionally, the high number of available GI-19 sequences from EU member states, along with the greater reliability of trade data and other variables considered in the study, was expected to enhance the sensitivity of the analysis.

### Phylodynamic analysis results

The time to most recent common ancestor (tMRCAs) for GI-16, GI-19, GI-23 HVR12 and GI-23 HVR3 were estimated in 1978.86 [95 High Posterior Density (95HPD):1943.90-1982.99], 1977. 92 [95HPD:1932.53-1991.72] 1984.02 [95HPD: 1955.32-1985.21] and 1969.44 [1937.57-1986.14]. The respective evolutionary rates were 6.23·10^−3^ [95HPD: 3.16·10^−3^ −9.88^−3^], 4.04·10^−3^ [95HPD: 2.90·10^−3^ −4.81^−3^], 6.92·10^−3^ [95HPD: 4.02·10^−3^ −9.36·10^−3^]. and 1.86·10^−3^ [95HPD: 1.41·10^−3^ −2.33^−3^].

For the European GI-19 dataset the tMRCA and evolutionary rate were 2000.90 [95HPD: 1979.69-2003.38] and 6.40·10^−3^ [95HPD: 4.73·10^−3^ −8.21·10^−3^].

**Fig. 1:**
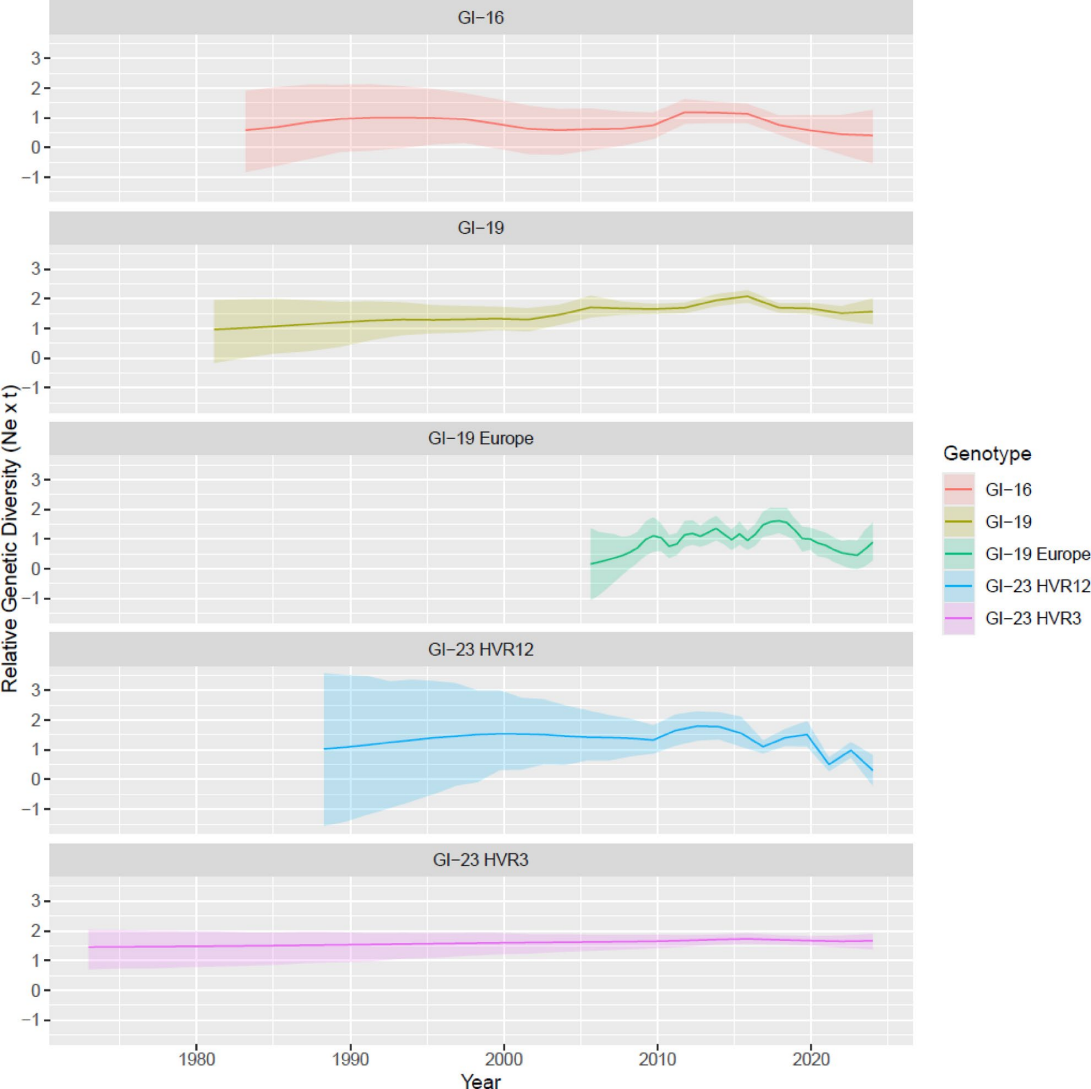
Estimated changes in effective population size over time for different IBV lineages, depicted in separate panels, based on Bayesian SkyGrid reconstruction. The mean estimates and their corresponding 95% highest posterior density (HPD) intervals are represented by solid lines and shaded areas, respectively. For each lineage, the mean value of the tMRCA has been selected as the starting point for the effective population size representation.

The reconstruction of viral population dynamics revealed both similarities and notable differences among the examined lineages. The GI-16 lineage exhibited a markedly different trajectory, characterized by a substantial rise in population size during the late 1980 s to late 1990 s, followed by a decline. A subsequent sharp resurgence was observed between 2010 and 2015, highlighting a distinct evolutionary dynamic compared to the other lineages (Fig. 1). Lineages GI-19 displayed a progressive but moderate increase in population size, which persisted until approximately the year 2000. This was followed by a more pronounced expansion phase through multiple “waves” lasting until around 2010–2015, after which population size appeared to stabilize. In Europe, after a sharper rise following the introduction, several waves were observed until a final overall decline starting from 2015 onward.

GI-23, especially when considering the HVR12 region, showed a progressive but moderate increase in population size after its emergence, which persisted approximately until 2000. This was followed by a phase of stabilization and a sharper increase after 2010. In this case as well, two additional major waves were estimated, although they exhibited a general decreasing trend. Overall, the same patter was estimated with the HVR3, although the resolution in population fluctuation was much lower (Fig. 1).

**Fig. 2:**
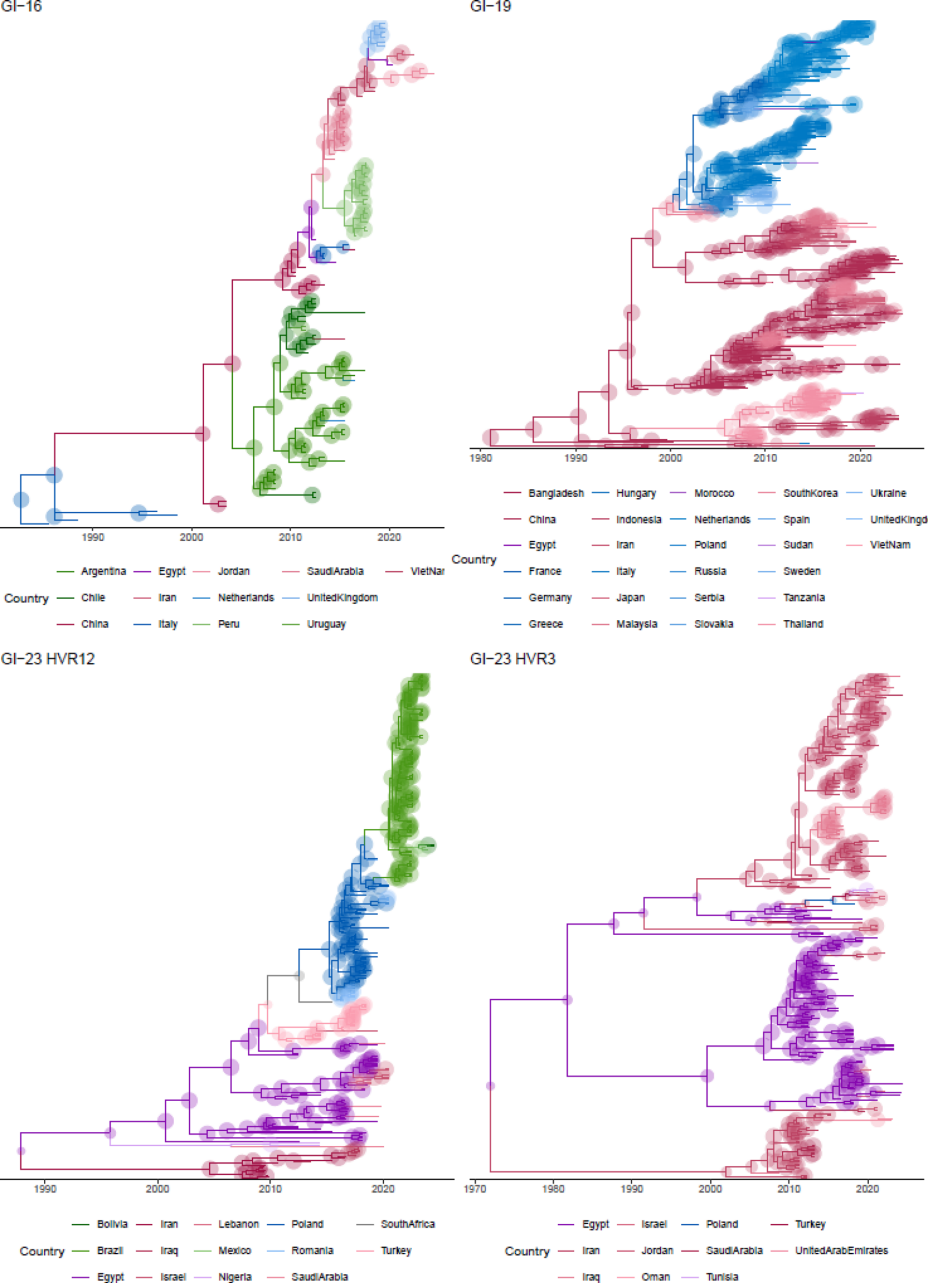
Time-scaled phylogenetic trees of IBV lineages GI-16, GI-19, GI-23 HVR12, and GI-23 HVR3 based on MCC tree reconstructions. Each panel shows the evolutionary history of the respective lineage, with time on the x-axis and tip nodes and branches coloured by macro-areas of origin. Individual countries within these areas are reported with different nuances. Circles size reflects the confidence in ancestral country estimation.

**Table 1:** Summary of the number, collection countries and period of the sequences included in the present study for different lineages/dataset.

Lineage	Seq. Number	Countries (Sequence number)	Time interval
GI-16	112	Argentina (28), Peru (17), Chile (12), Saudi Arabia (12), China (10), Italy (7), United Kingdom (6), Vietnam (6), Jordan (4), Egypt (3), Iran (3), Netherlands (2), Uruguay (2)	1983–2022
GI-19 (Subset)	626	Bangladesh (1), China (192), Egypt (1), France (4), Germany (28), Greece (2), Hungary (3), Indonesia (23), Iran (14), Italy (101), Japan (12), Malaysia (16), Morocco (1), Netherlands (104), Poland (11), Russia (1), Serbia (2), Slovakia (2), South Korea (35), Spain (5), Sudan (1), Sweden (9), Tanzania (1), Thailand (47), Ukraine (3), United Kingdom (1), Vietnam (6)	1993–2023
GI-23 HVR3	271	Egypt (93), Iran (112), Iraq (26), Israel (12), Jordan (9), Oman (1), Poland (1), Saudi Arabia (11), Tunisia (2), Turkey (2), United Arab Emirates (2)	1998–2024
GI-23-HVR12	298	Bolivia (2), Brazil (117), Egypt (48), Iran (13), Iraq (6), Israel (6), Lebanon (3), Mexico (2), Nigeria (2), Poland (60), Romania (12), Saudi Arabia (4), South Africa (1), Turkey (22)	1998–2024
GI-19 Europe	458	France (4), Germany (28), Greece (2), Hungary (3), Italy (185), Netherlands (203), Poland (11), Russia (1), Serbia (2), Slovakia (2), Spain (5), Sweden (9), Ukraine (3).	2004–2020

### Phylogeographic analysis

**Fig. 3:**
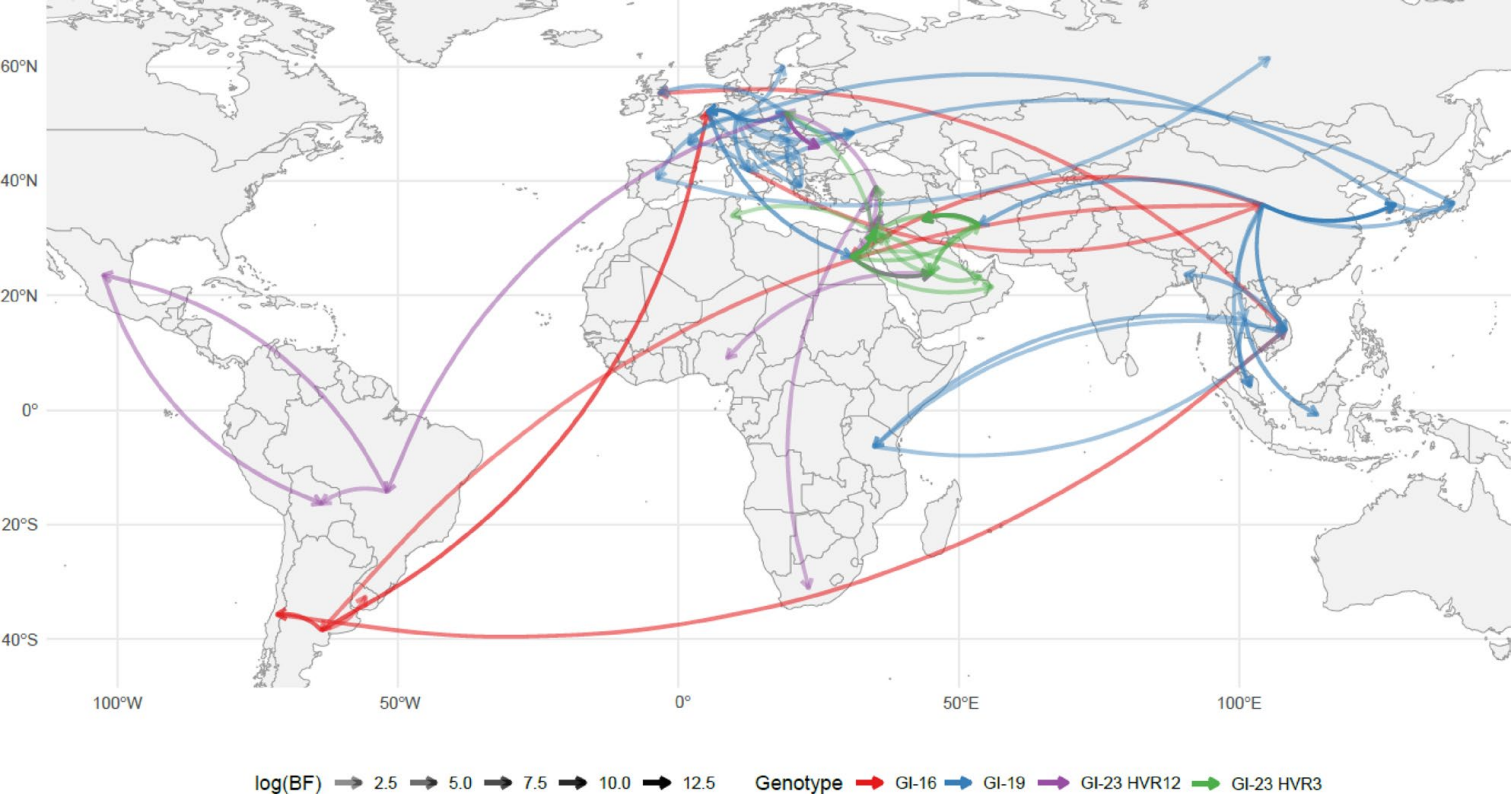
Bayesian reconstruction of IBV migration routes by IBV lineage (colour coded). The map illustrates statistically supported viral migration events inferred for IBV lineages GI-16 (red), GI-19 (blue), GI-23_HVR12 (purple) and GI-23_HVR3 (green). Arrows indicate the direction of viral movement between countries, with line color saturation proportional to the strength of statistical support (log Bayes Factor). A more detailed, lineage-specific, depiction is provided in supplementary Fig. 1.

The phylogeographic reconstruction of GI-16 migration revealed a progressive and widespread dispersal (Fig. 2), with several well-supported migration events connecting Europe, Asia, and South America (Fig. 3). Following a likely European origin, the lineage appears to have spread to China around 2005, subsequently reaching South America (approximately in 2010), where it became established after local circulation, and the Middle East around 2015. A reintroduction in Europe also occurred around the same time. Additionally, a more recent introduction in Peru may have originated from the Middle East (Fig. 2), although this specific migration pathway lacks strong statistical support, and an Asian origin remains similarly plausible. Notably, one Dutch strain clustered closely with Argentinian sequences, suggesting contacts between Europe and South America. It is important to note that the limited number of available sequences from certain regions may have affected the robustness of phylogeographic inferences. Notably, Asia appears to have played a role in the dissemination of viral strains to both North and South America, as well as Europe. This may lead to spurious associations in the reconstruction of migration pathways. For example, the apparent introduction of strains from Argentina to Europe might instead reflect independent introductions of genetically similar Chinese strains into both regions. Such a pattern could create the illusion of a direct South America–Europe connection, while the true source may lie elsewhere.

The origin of the GI-19 lineage was inferred in Asia, most likely in China, where it circulated for decades before spreading southward and eastward to other Asian countries around the year 2000. A nearly simultaneous introduction in Europe was also detected, approximately around 2003. Our analysis suggests that, from the initial entry point in Southern Europe (notably Italy), the virus underwent centrifugal dispersal to other European countries, including Mediterranean nations such as Spain and Greece and central Europe, as Poland and Germany, and further northward. In Europe, GI-19 has exhibited widespread and sustained circulation from its initial introduction at the beginning of the millennium up to the present, forming an independent and well-supported European cluster. The introduction of GI-19 into Northern African countries, that was estimated to begin in the first decade of the new millennium, appears to be primarily linked to European sources. However, migration events among African countries were also inferred, suggesting regional circulation of the virus. In contrast, the introduction of GI-19 in Central African countries seems to have occurred more recently, around 2020, likely originating from Asian countries (Figs. 2 and 3).

Combining the results obtained from the GI-23 HVR12 and GI-23 HVR3 datasets, the virus appears to have circulated persistently in the Middle East from its emergence until approximately 2010. Subsequently, it underwent progressive geographic expansion, particularly affecting Eastern Europe—likely through Turkey—and later extending to Central and Southern Africa (~ 2011–2013), and more recently to Northern Africa (~ 2017). The introduction in South and Central America occurred around 2018, likely through two independent introduction events from Europe, potentially Poland. From there, the virus spread to Mexico, which may have facilitated its introduction in Bolivia, although a direct route from Brazil to Bolivia was also significantly supported (Fig. 3). Strains detected in Asia, as well as some American ones, showed a close relationship with vaccine strains.

### Determinants of viral dispersal

**Fig. 4:**
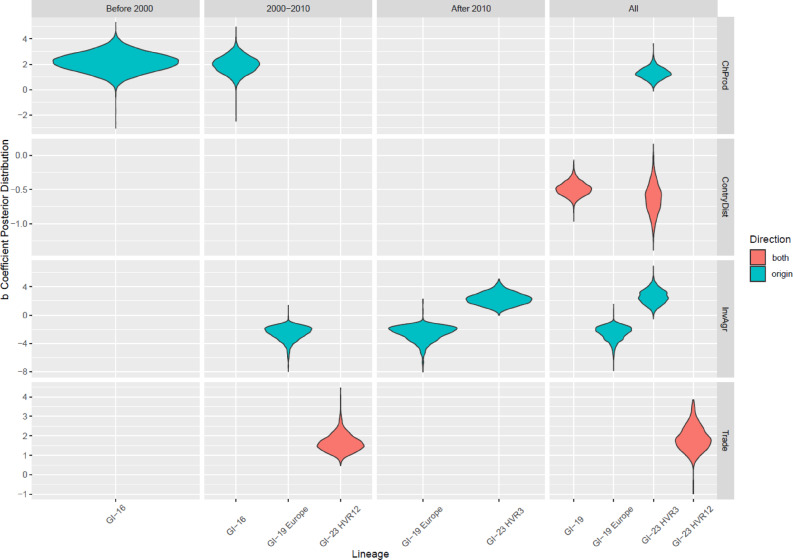
Violin plots showing the 95HPD distribution of estimated b coefficients relating viral migration rates and potential explanatory variables across different IBV genotypes and time periods. Each panel represents a variable potentially influencing viral spread: ChProd (chicken production), CountryDist (geographical distance among countries), InvAgr (investments in agriculture), and Trade (live poultry trade). Genotypes are shown along the x-axis, with values of association (y-axis) stratified by period: before 2000, 2000–2010, after 2010, and overall (All). Colors indicate the site for which the predicted were significantly associated (Origin = Country of origin of viral dispersal; Both = Both countries).

Among the factors evaluated as potential predictors of lineage migration rates, only a few showed statistically significant associations (Fig. 4). A significant positive correlation with chicken population size was observed for GI-16 prior to 2000 and between 2000 and 2010, as well as for GI-23 across the entire study period. Conversely, a negative association with inter-country distance was identified for both GI-19 and GI-23 when the whole time period was considered.

The impact of agricultural investment exhibited a lineage-dependent pattern: a negative association was found for GI-19 in Europe from 2000 onward and when considering the average over the entire period, whereas a positive association was detected for the GI-23 lineage after 2010 and for the full study duration. Similarly, a statistically significant positive association with chicken trade among countries was observed for the GI-23 lineage only, particularly when the HVR12 was considered.

## Discussion

The epidemiology of different IBV variants and lineages has been described in several studies. However, most investigations have focused on limited geographic areas. Even when contextualized within an international scenario, analyses are mainly based on phylogenetic comparisons, often lacking a formal statistical framework^25^, with an arbitrarily selected set of reference sequences, which can introduce bias driven by researchers’ prior beliefs and pre-set hypotheses.

In contrast, the present study aimed to assess the history, epidemiology, dispersal patterns, and determinants of the major IBV lineages by including all publicly available sequences, selected according to formal and pre-established criteria. This strategy was specifically designed to minimize subjective assumptions and ensure a more objective and reproducible evaluation of viral dynamics at a global scale.

The selected lineages also represent both historical and current examples of major threats to the poultry industry, that were perceived as critical by veterinarians and farmers. In particular, the GI-16 lineage (also known as 624/I) was retrospectively shown to have circulated widely in Italy since the early 1960 s, in agreement with the tMRCA 95% HPD herein estimated. It remained the most prevalent genotype in Italy for over a decade before expanding to Asia. Starting in the early 2000 s, the GI-19 lineage emerged as the dominant and more concerning variant, maintaining its relevance for the following decades^4^. More recently, attention from stakeholders has increasingly shifted toward GI-23, whose rapid expansion has raised significant concern^26^.

However, the evaluation of tMRCA and population dynamics revealed a substantial overlap in the origin of these genotypes, dating back to the 1960–1980 s. This suggests that what appears—or is perceived—as the progressive emergence of new variants is, in fact, the result of ancient variants or lineages that circulated largely undetected at low levels for extended periods. Their apparent emergence as major threats is likely driven by changes in environmental or epidemiological conditions that favour their expansion^27^. The introduction in new regions—characterized, for example, by lower immunity or specific farming practices—may act as a key trigger of such emergence. This pattern was observed with the GI-16 lineage, which, after being introduced in Asia, experienced a marked increase in circulation, followed by a decline. A similar, although directionally opposite, dynamic was observed for GI-19. This lineage circulated persistently in Asia with a slow but steady increase, likely linked to the intensification of poultry production^4^. However, a major rise in prevalence was observed following the introduction of the virus in Europe. This pattern, evident in the overall GI-19 population, became even more pronounced when European GI-19 strains were analyzed independently. The introduction in the naïve European poultry population was followed by a marked increase, characterized by successive waves of viral expansion corresponding to the expansion into new EU countries. The situation was eventually brought under control through improvements in vaccination protocols—first in Europe, and subsequently in Asia as well, as previously reported (Franzo et al., 2024). Interestingly, the decreasing phase of GI-16 overlaps with the rise (around 2000) of the main co-circulating lineage, GI-19. GI-16 may have initially suffered from competitive exclusion by the dominant GI-19 lineage and subsequently from the implementation of more effective control measures. These measures, prompted by the severe clinical signs and economic losses caused by GI-19, included the extensive adoption and refinement of vaccination strategies—both homologous and heterologous (mainly GI-1 based vaccination combined with GI-13 based vaccines)—which were also shown to be effective against GI-16^28^. The last GI-16 rise observed, on the other hand, reflects the introduction of the lineage in South America (~ 2005), where a naïve niche was likely found and where several countries were rapidly affected including Argentina, Chile, Colombia, Peru, and Uruguay^29^. This pattern was likely further facilitated by differences in vaccination approaches, as most South American countries rely primarily on GI-1-type strains in their IBV vaccination programs. Only recently has the use of other strain types (e.g., 793/B (GI-13) or BR1 (GI-11)) been officially authorized^30^, typically in combination with GI-1, to provide high levels of protection against heterologous strains^31^. However, the use of a single, heterologous vaccine, is still applied in some countries as Peru, and might have contributed to the persistent circulation of GI-16 in these areas.

The pattern observed for the GI-23 lineage is slightly more nuanced, though still consistent with previous observations. A progressive increase in viral population size was noted, likely reflecting the gradual and less abrupt spread from Middle Eastern countries to neighbouring regions^19,26^. Rise in the viral population were observed reflecting the introduction to Europe, African and ultimately American countries, where GI-23 was able to persistently circulate. The temporal trends in IBV lineage frequencies reported in other South American studies appear to support this model. Specifically, the GI-11 lineage initially predominated but showed a marked decline in relative frequency by 2022, coinciding with the emergence of GI-23, which maintained a substantial presence through 2024^29^, reflecting Skygrid reconstruction and confirming the likely competition among co-circulating lineages. The introduction and maintenance of new variants, causing significant clinical signs and production losses, has highlighted the fragility of the South American poultry system to such events. This has prompted intense discussion on the introduction of new vaccines based on different strains, a strategy that has only recently begun to be implemented. The emergence of GI-23 has further reinforced the urgency of updating vaccination schemes, although the use of homologous vaccines is not yet officially authorized^17,30,32^. Nevertheless, some American strains were found closely related to vaccine strains. This raises the possibility that modified live vaccines—either directly or via vaccinated animals—may have been improperly introduced in the region, potentially through unauthorized use and thereafter inadequate handling. Such mistakes in vaccine management could have allowed these strains to persist, evolve, and spread—an occurrence repeatedly reported for IBV and other modified live vaccines^33,34^.

Therefore, greater efforts are needed to understand the source of these introductions and to assess the actual benefits that could result from widespread vaccine use in the region—particularly if not properly planned, administered and controlled.

Overall, these observations further underscore the dynamic nature of IBV epidemiology, where shifts in lineage predominance occur over time, primarily driven by the introduction of new variants and the use of vaccines. Clearly, extrapolating overall global patterns inevitably involves a degree of oversimplification, as substantial variability can be expected at the national level, and even among individual companies or farms. However, the evidence from this study suggests that IBV lineages should not be regarded as entirely independent entities. Their epidemiological success may influence the dynamics and fate of other co-circulating variants, through complex interactions in regions where their distribution overlaps. For this reason, understanding their spreading patterns and potential is of critical importance.

The IBV lineages considered in this study seems to demonstrate remarkable spreading potential and highly variable dispersal patterns. Based on the phylogeographic reconstruction, in the Old World, viral flow occurred both from west to east—such as the initial spread of GI-16 and, to a lesser extent, GI-19 during its later migration phase—and from east to west, as in the early spread of GI-19 and the subsequent re-expansion of GI-16^35^. GI-23, on the other hand, apparently followed a centrifugal dispersal pattern, spreading to Europe, Asia, and Western African countries.

Africa represented a notable exception, acting predominantly as a strain importer from Asia, Europe, and the Middle East. Interestingly, the source of these strains broadly mirrored—albeit in a simplified manner—the spheres of influence of the three macro-regions: Asia exported primarily to Central-Western Africa, Europe to Northern Africa, and the Middle East to neighboring northern areas. Nevertheless, the evidence of intra-African circulation was also observed.

While long-distance transmission events often attract the most attention, they were relatively rare. All lineages—particularly GI-19 and GI-23, for which the greater number of available sequences allowed higher analytical resolution—demonstrated stable local persistence at country level and a much denser network of local transmission events. The web of statistically supported migration rates among Central and Southeast Asian countries, as well as among European countries for GI-19, and among Middle Eastern countries for GI-23, was significantly denser compared to intercontinental migration patterns.

In fact, among the factors assessed using the GLM, geographic distance between countries was found to be a significant negative predictor of viral migration rates for both genotypes. The role of a country in viral dissemination appears more closely linked to the characteristics of the local host population. Among the factors considered, chicken population size emerged as the primary variable positively correlating with migration rates: the larger the host population in the country of origin, the higher the probability of viral exportation—just as more sparks are likely to emerge from a large fire. In particular, the relationship was statistically significant before 2010 for GI-16 corresponding to the periods of major geographic dispersal—and throughout the entire study period for GI-23, which showed a more progressive worldwide expansion over the whole considered time period.

Surprisingly, the effect of trade was not statistically significant in most instances, although an overall positive correlation between poultry trade volumes and viral migration rates was observed (data not shown). While this result may seem unexpected, it can be explained by the IBV epidemiological scenario, which—potentially also due to the virus’s lability— is primarily characterized by within-country persistence and circulation, with only occasional cross-border—and even less frequent intercontinental—migration events.

To further explore this aspect, the European Union was specifically considered, as the absence of trade restrictions should theoretically facilitate inter-country exchanges. Additionally, the high number of GI-19 sequences from EU member states, along with the greater availability of trade data and other variables considered in the study, was expected to enhance the sensitivity of the analysis. Nevertheless, even in this context, trade was not a significant driver of viral spread, and strong geographical clustering remained evident. Therefore, although trade was clearly biologically and epidemiologically relevant in certain introduction events, based on our results the movement of infected animals or contaminated materials proved to be only sporadically effective. The only exception was represented by GI-23 when HVR12, available for a higher number of countries, was considered. In this case, a positive effect of viral migration rate was observed. This finding may be linked to the different socio-economic context in which this variant initially circulated—namely, a production system that, although growing in terms of capacity, had not yet developed the necessary tools to effectively control the infection. These include, among others, the presence of adequate protocols for animal trade management aimed at minimizing the risk of strain introduction from foreign countries. Moreover, expanding farming system might be more at risk of introducing viruses through embryonated eggs and live bird importation for supporting production or genetic lines improvement.

Consistent with this interpretation, agricultural investment within Europe showed a strong negative correlation with viral migration rates. Although this is an aggregate measure encompassing various aspects, improvements in biosecurity, diagnostics, and the implementation of more refined and widely adopted vaccination strategies likely played a central role, either directly or indirectly. Such measures have repeatedly been shown to limit viral circulation at both local and regional levels, thereby reducing the risk of cross-border transmission. A previous study demonstrated the effectiveness of proper vaccination strategies against GI-19 in Europe compared to Asia, particularly during the earlier phases of the lineage spread, although this gap has progressively narrowed over the last decade^36^. On the other hand, the positive effect of investments in agriculture detected for GI-23 likely reflect the notable expansion of the poultry sector in Middle Eastern countries, that has required increased investments in agriculture and has likely resulted in more frequent interactions with neighbouring countries. However, this growth has not been adequately accompanied by corresponding improvements in animal health management and infectious disease control. Unfortunately, in several countries, control strategies—particularly vaccination—are still driven by subjective perceptions, the influence of opinion leaders, or market trends, rather than by a sound understanding of the local epidemiological context. This often results in fragmented and inconsistent interventions, which may be largely ineffective or even counterproductive. From a statistical perspective, the true association between an expanding—but still poorly managed—poultry sector and viral dissemination, could thus contribute to a spurious correlation between agricultural investment and increased viral migration.

One of the most intriguing findings concerned the introduction of IBV lineages in the Americas. In both cases, multiple—albeit limited—introduction events appear to have occurred, especially from Asia for GI-16 and from Europe for GI-23. Both lineages established significant local circulation, spreading across countries and demonstrating persistence and expansion within the continent. Understanding the mechanisms behind these introductions remains challenging, especially given the scarcity of properly documented chicken trade between the source and recipient regions. This suggests the involvement of alternative, less obvious transmission pathways that warrant further investigation. The role of migratory birds in IBV dissemination has been proposed by several authors, primarily based on their documented susceptibility to coronavirus infections. However, such detections are rare, and in most cases, identification is based on diagnostic assays or partial genome sequencing targeting regions that allow classification at species level with limited confidence, without confirming actual IBV infection. Moreover, the detection frequency is generally low and the ability of migratory birds to shed the virus and their true epidemiological relevance remain unknown^37–39^.

A recent study suggested the existence of clusters of IBV strains with distribution patterns seemingly aligned with migratory flyways. The limited number and arbitrary selection of sequences (~ 300, over 200 of which were references from Valastro et al., 2016), combined with the lack of any formal modelling or statistical support poses concerns on the strength of this claim^22^.

In contrast, the present study analyzed hundreds to thousands of sequences per lineage, collected across dozens of countries and over multiple decades. These data were assessed within a dedicated statistical framework designed to evaluate the association between viral migration and shared migratory flyways. No evidence was found to support a relevant role for migratory birds in IBV spread. While this does not entirely exclude the possibility that migratory birds may sporadically mediate IBV transmission, the findings support the conclusion that their role is negligible from an epidemiological standpoint.

Specifically, regarding the introduction of IBV into the Americas, additional objections may be raised. Notably, the migration of avian influenza virus (AIV) from Eastern Asia to North America has been reported on multiple occasions and is thought to be mediated by migratory birds with overlapping flyways^40–42^. The East Asian and Pacific American migratory routes intersect over Eastern Russia and Alaska, particularly around the Bering Strait. It is estimated that 1.5 to 3 million aquatic birds migrate from Asia to Alaska annually during the breeding season, providing a substantial potential route for viral dissemination^43,44^. Similarly, the role of the Atlantic Rim in facilitating viral introduction from Europe to North America via wild bird migration has recently been proposed for AIV^45^ even if such events appear to be relatively infrequent. Furthermore, no major migratory routes connect Asia, Europe, or Africa directly to South America. Therefore, based on flyways structure, if migratory birds were indeed involved, an earlier introduction in North American countries would be more plausible than a direct entry into Central or South America. While the possibility of undetected infections cannot be completely ruled out, the evidence supporting multiple independent introduction events weakens this hypothesis. Moreover, if flyways were involved, the introduction of the GI-19 genotype—widely circulating in both Europe and Asia—would also have been expected, which was not observed.

Based on this evidence, alternative sources of introduction should be taken into account, even though each of them—when considered individually—appears to carry a relatively low likelihood. The legal importation of live poultry or contaminated fomites is unlikely to have been a major driver, as such imports are limited and primarily occur between other American countries, among which actual strain exchange has been demonstrated. Illegal importation cannot be entirely excluded, although live bird or raw poultry product smuggling poses substantial logistical and sanitary challenges due to current transportation and biosecurity regulations. Fomites (feed/slaughter contacts) and human movements (company technician, veterinarian, shared farm workers) could also be involved^17^.

Overall, the study highlights the complex, lineage-specific nature of IBV spread and underscores the need for science-based, well-coordinated control strategies supported by international surveillance and sustained investments in poultry health management.

In light of this evidence, future efforts should focus on the development of a comprehensive and integrated surveillance strategy, which includes prompt diagnosis and effective monitoring systems. This should not only target official trade routes but also account for human movements, shared equipment, and potential indirect transmission pathways, in order to identify and mitigate even low-likelihood sources of viral introduction.

## Materials and methods

### Dataset Preparation

Given the extensive genetic diversity and ancient origin of IBV, reconstructing the complete evolutionary history and global migration dynamics at the species level is currently difficult due to the high uncertainty surrounding ancient spreading events. Therefore, the analysis was conducted at the lineage level. To ensure computational feasibility and facilitate result interpretation, a limited number of lineages were selected as case studies based on specific inclusion criteria. Lineages had to exhibit international distribution, involving multiple countries across different continents. Moreover, to avoid confounding effects, lineages for which modified live vaccines were available were excluded unless vaccine-derived strains were clearly distinguished from field strains based on sequence analysis. Additionally, a sufficiently large number of sequences (i.e., > 100) with known collection country and date was required for each lineage, following the exclusion of low-quality sequences and those not matching the selected genomic region or other inclusion criteria.

Initially, all complete and partial IBV spike protein sequences were downloaded from GenBank and classified into genotypes and lineages by comparison with the references proposed by Valastro et al.^8^. To this end, the reference sequence dataset was retrieved and aligned. Subsequently, using in-house developed Python scripts, all downloaded sequences were iteratively aligned to the reference sequences one by one using MAFFT^46^.

A sequence was assigned to a given lineage if its genetic distance from at least one of the reference sequences was below 13%. Independent datasets were then generated for each lineage. Since different laboratories and research groups target various regions of the spike protein gene, all sequences assigned to a lineage were aligned, and the region of the alignment offering the best trade-off between sequence length and number of available sequences was selected and only sequences having at least a 90% coverage of the region were included for further analysis. For genotypes for which live vaccines have been marketed, strains sharing a percentage of identity higher than 99% with a reference vaccine sequence were excluded from the analysis^47,48^. Moreover, the inclusion of recombinant strains is highly detrimental to the reliability of phylogenetic and phylogeographic inferences, thus warranting their removal^49^. Unfortunately, due to the high number of sequences—in the order of thousands for certain lineages—commonly applied recombination detection methods encounter computational and interpretative limitations. Therefore, a preliminary screening was conducted using an in-house developed Python script.

Briefly, for each lineage, selected sequences were aligned to the reference sequences, and pairwise genetic distances were calculated using a sliding window approach (windows of 200 nt, sliding by 20 nt). Only sequences that consistently had a member of the same lineage as their closest relative across all windows were retained in the dataset; the others were discarded as potential recombinants.

Following this preliminary filtering, the tentative non-recombinant dataset was further evaluated for recombination signals using RDP5^50^ and GARD^51^ to confirm the absence of detectable recombination events.

### Viral population dynamics and phylogeography

Population parameters of the selected IBV lineages —including the time to the most recent common ancestor (tMRCA), evolutionary rate, and temporal variation in population dynamics—were jointly estimated across five independent datasets using the Bayesian serial coalescent approach implemented in BEAST vX10.5.0^52^. The most appropriate nucleotide substitution model (i.e. GTR + G4 + I) was identified using the Bayesian Information Criterion (BIC), as calculated by JModelTest2^53^. The optimal molecular clock model was selected based on Bayes Factor (BF) comparisons, obtained by estimating marginal likelihoods through path sampling (PS) and stepping-stone (SS) methods^54^. To infer changes in relative genetic diversity over time (i.e., effective population size × generation time, Ne × t), the nonparametric Skygrid model was employed^55^. Strain migration among countries was reconstructed using the asymmetric discrete-state phylogeographic model described by Lemey et al.^56^, treating each country of sample collection as a discrete trait. This framework allows trait mapping along a time-calibrated phylogeny under a molecular clock model, while incorporating changes in population size and accounting for phylogenetic uncertainty.

The Bayesian Stochastic Search Variable Selection (BSSVS) procedure was included to identify the most parsimonious phylogeographic diffusion pathways via Bayes Factor testing. All parameters were estimated jointly in a Bayesian framework using a Markov Chain Monte Carlo (MCMC) chain of 200 million generations, with sampling every 20,000 steps. Convergence and sampling efficiency were assessed using Tracer v1.7, after discarding the initial 20% of samples as burn-in. Only runs with an effective sample size (ESS) > 200 and adequate trace mixing, verified by visual inspection, were retained for further analysis.

Maximum clade credibility (MCC) trees were generated using TreeAnnotator (part of the BEAST suite), summarizing the posterior distribution of trees. Spatiotemporal viral diffusion across Europe was visualized using SPREAD3^57^. Migration routes between countries were considered statistically supported if the corresponding BF exceeded 10.

### Evaluation of factors involved in IBV lineage dispersal

The relevance of different variables in affecting the viral migration rate was investigated using a generalized linear model extension of phylogenetic diffusion to perform Bayesian model averaging over candidate predictors^25,58^. In the framework of Bayesian discrete state phylogeographic approach, the rates at which viruses transition between pairs of countries are typically denoted as the ij*th* elements (Λij) of a K·K transition rate matrix, being K the number of involved countries. Such rates can be parameterized as a log-linear function of various potential predictors (p) using a generalized linear model (GLM)^58^ where each potential predictor in the GLM is associated with a β_p_ coefficient, quantifying its effect size on Λ, and a binary indicator δ_p_ that determines its inclusion or exclusion of the predictor in the model:

log Λij = log (p1) × *β*1 × *δ*1 + log (p2) × *β*2 × *δ*2 +⋯+log (pn) × *β*n × *δ*n.

The δ variables are estimated using a BSSVS^59^, resulting in an estimate of the posterior inclusion probability or support for each predictor. The BF was calculated as the ratio of the posterior odds over the prior odds for predictor inclusion and used to express the formal inclusion support of a variable. In the present study, a Bernoulli prior probability distribution was set on these indicators, assigning a 50% prior probability on no predictors being included in the model. A predictor was considered statistically supported if BF > 10.

Several potential predictors of viral dispersal were considered, including animal population (chicken population size and trade), economy (per capita income, investments in agriculture, share of people working in agriculture), and geographical (minimum distance between country borders). Data were obtained from COMTRADE (https://comtradeplus.un.org/), FAOSTAT (https://www.fao.org/faostat/en/) and EUROSTAT (https://ec.europa.eu/eurostat) archives (last accessed on 20/12/2024) or from country-specific databases when information was missing. These variables were summarized as the overall mean across all available years, as well as for three specific time periods: before 2000, between 2000 and 2010, and after 2010.

Pairwise minimum distances between country borders were calculated using a custom Python script. Shapefiles of country geometries (Natural Earth, 1:110 m scale; https://www.naturalearthdata.com/) were processed with GeoPandas^60^, and the minimum boundary-to-boundary distance between each pair of countries was computed using Shapely geometry operations.

The potential involvement of migratory birds was also evaluated. For this purpose, images representing the areas covered by major migratory flyways were downloaded from datazone-birdlife (https://datazone.birdlife.org/). As the images were in a different projection system than the country shapefile, they were imported into QGIS^61^ as raster layers and georeferenced to the shapefile by manually matching multiple geographic landmarks across both maps. The raster images were then reprojected to a common coordinate reference system, enabling spatial overlay and automatic identification of countries intersected by each migratory flyway. A georeferenced raster image depicting migratory flyway areas was processed to extract the corresponding geographic coverage. A binary mask was generated by isolating pixels within a defined colour range corresponding to the flyway region. After morphological erosion to refine borders, the identified regions were converted into vector polygons using affine transformation. These were then intersected with the world map, and countries with an overlap greater than 10% of their surface area were retained.

The aforementioned potential predictors of migratory rates were integrated into the phylogeographic GLM framework, after filtering for countries (or country pairs) for which sequence data were available for the lineage under investigation.

Briefly, quantitative country-specific variables (e.g., chicken population size, agricultural investments, proportion of the population employed in agriculture, etc.) were log-transformed and standardized, and their effects were assessed for both origin and destination countries. Similarly, quantitative matrices involving country pairs (e.g., trade volumes or geographic distances, etc.) were log-transformed and standardized. In both cases, zero values were replaced with 0.001 prior to log transformation to avoid undefined values. Finally, qualitative predictors (e.g., whether two countries are intersected by the same migratory bird flyway) were coded as binary variables (0 = “No”, 1 = “Yes”) and included without further transformation. Predictors for which no variability was present in the specific dataset were excluded.

The contribution or effect size of each significantly supported predictor was estimated by summarizing the posterior distribution of the associated regression coefficient (β). It is important to note that these estimates are conditional on the inclusion of the predictor in the model, as determined by its corresponding binary indicator variable (*δ*). Specifically, when the indicator equals 1, the predictor is included in the model and the coefficient is informed by the data (i.e., the predictor values and the phylogeographic state transitions). Conversely, when the indicator equals 0, the predictor is excluded and the coefficient is sampled from the prior distribution. Therefore, the reported effect sizes represent conditional estimates, calculated by summarizing only those samples in which the predictor was included in the model (i.e., *δ* = 1).

## Supplementary Information

Below is the link to the electronic supplementary material.

Supplementary Material 1

## Data Availability

All sequences produced in the study are freely available in GenBank.
