# Supplementary figures and images for: Phylodynamic and phylogeographic reconstruction of IBV lineages: diverse paths and determinants, one goal for control
Source: Sci Rep. 2025 Oct 23;15:37068. doi: 10.1038/s41598-025-21138-8 (PMC12550103; doi:10.1038/s41598-025-21138-8)

GI16

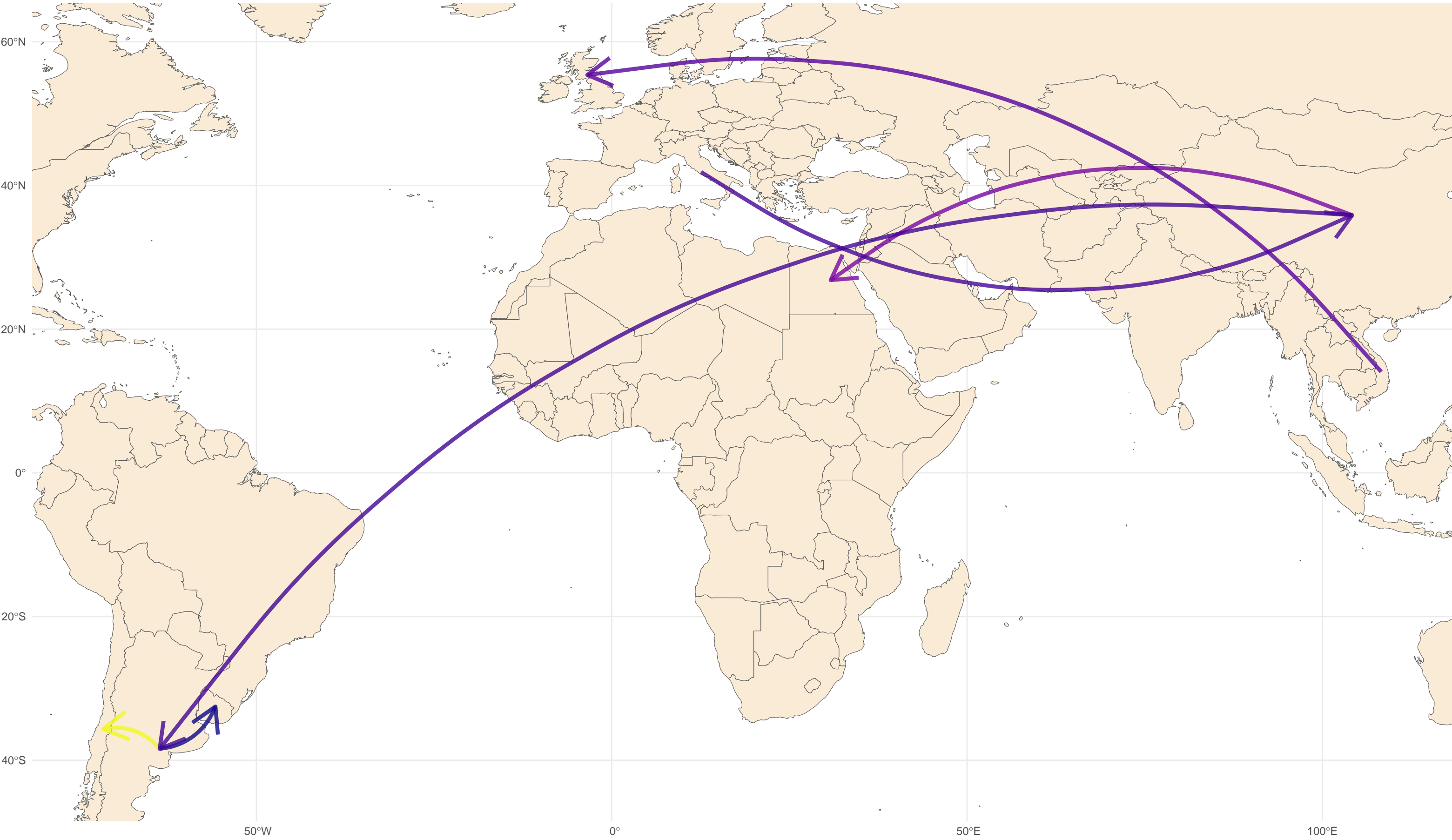

GI19

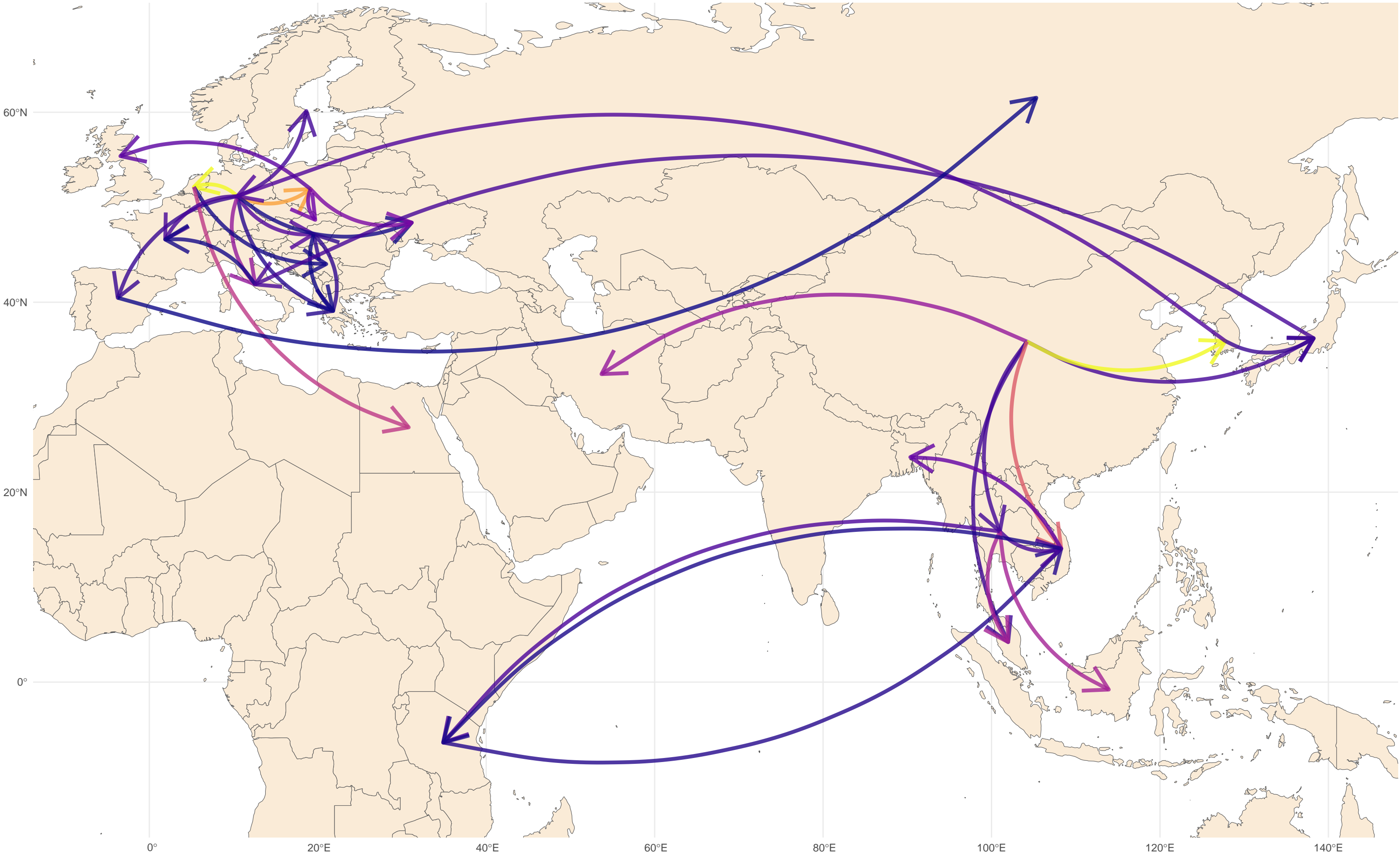

GI23-HVR12

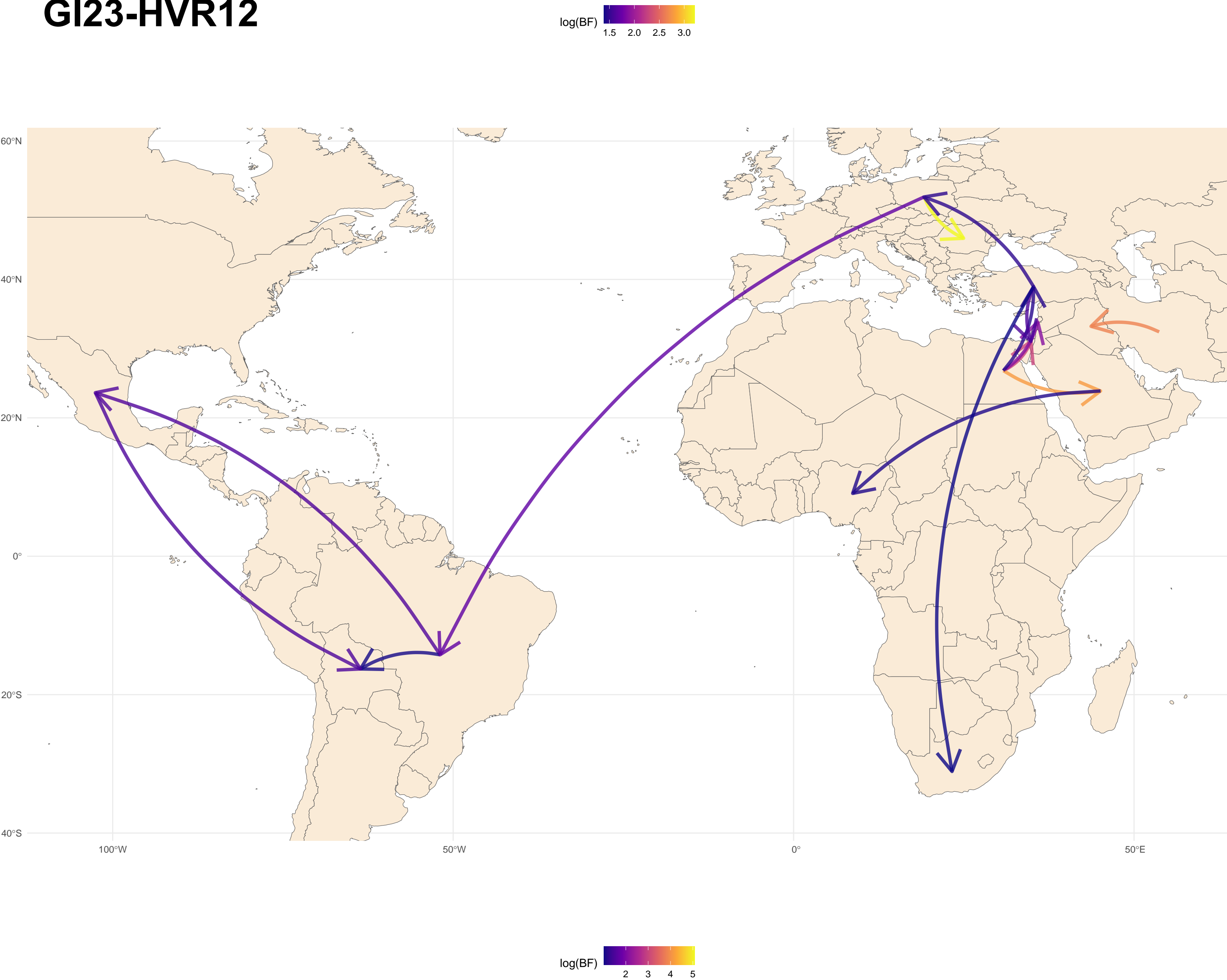

GI23-HVR3

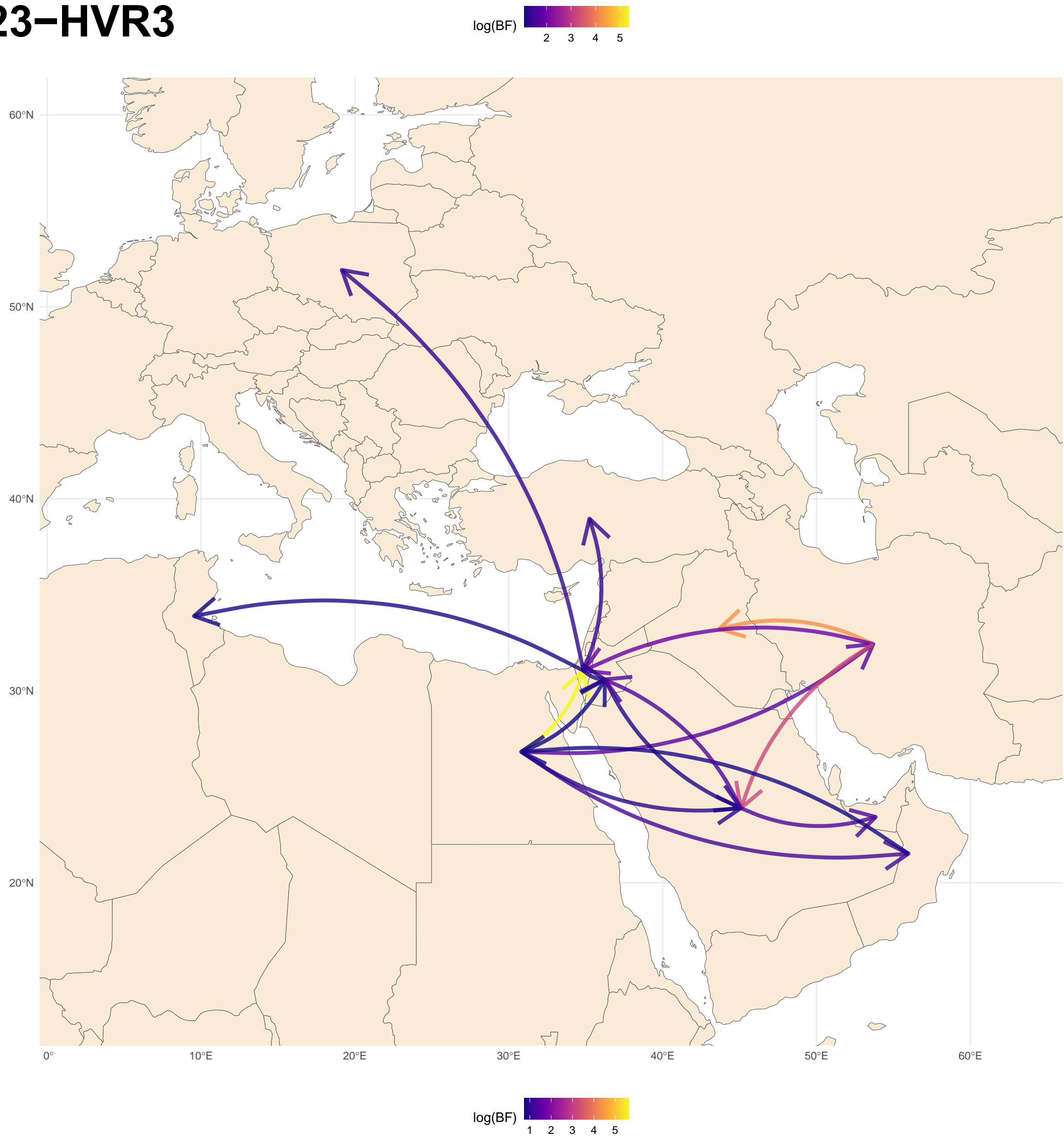

Supplement: Supplementary file 1 — Supplementary Material 1 [file 41598_2025_21138_MOESM1_ESM.pdf]
